# Supplementary material for: GUY1 confers complete female lethality and is a strong candidate for a male-determining factor in Anopheles stephensi
Source: eLife. 2016 Sep 20;5:e19281. doi: 10.7554/eLife.19281 (PMC5061544; doi:10.7554/eLife.19281)
Supplement: Figure 3—source data 1. — The mating strategy and progeny genotypes are described in Figure 3A. DOI: http://dx.doi.org/10.7554/eLife.19281.008 [file elife-19281-fig3-data1.docx]

**Figure 3-source data 1.** Number of the four types of progeny from wild type males mated with *nGuy1-1* (DsRed) and CFP positive males. These are source data for Figure 3*. The mating strategy and progeny genotypes are described in Figure 3A.

| L4 (tally) | CFP | DsRed | Both | None | | L4 (%) | CFP (%) | DsRed (%) | Both (%) | None (%) |
| --- | --- | --- | --- | --- | --- | --- | --- | --- | --- | --- |
| Replicate 1 | 25 | 31 | 0 | 40 | | Replicate 1 | 26.0 | 32.3 | 0.00 | 41.7 |
| Replicate 2 | 163 | 186 | 0 | 176 | | Replicate 2 | 31.0 | 35.4 | 0.00 | 33.5 |
| Replicate 3 | 17 | 13 | 0 | 17 | | Replicate 3 | 36.2 | 27.7 | 0.00 | 36.2 |
| Replicate 4 | 51 | 70 | 0 | 67 | | Replicate 4 | 27.1 | 37.2 | 0.00 | 35.6 |
| Total | 256 | **300** | 0 | 300 | | Average | 30.10 | 33.15 | 0.00 | 36.75 |
|  |  |  |  |  |  | |  |  |  |  |
|  |  |  |  |  |  | |  |  |  |  |
| L1 (tally) | CFP | DsRed | Both** | None | | L1 (%) | CFP (%) | DsRed (%) | Both (%) | None (%) |
| Replicate 1 | 32 | 35 | 2 | 30 | | Replicate 1 | 32.3 | 35.4 | 0.02 | 30.3 |
| Replicate 2 | 17 | 22 | 1 | 17 | | Replicate 2 | 29.8 | 38.6 | 0.02 | 29.8 |
| Replicate 3 | 64 | 72 | 8 | 75 | | Replicate 3 | 29.2 | 32.9 | 0.04 | 34.2 |
| Replicate 4 | 96 | 74 | 11 | 84 | | Replicate 4 | 36.2 | 27.9 | 0.04 | 31.7 |
| Total | 209 | 203 | 22 | 206 | | Average | 31.90 | 33.69 | 0.03 | 31.52 |

Notes:

* These are the numbers and percentages used in Figure 3. L4, screened at the L4 instar stage. L1, screened at the L1 instar stage. The total number (tally) and percentages (%) are shown separately.

**All double positives L1 larvae died prior to 8 hrs after hatch.
