# Supplementary material for: GUY1 confers complete female lethality and is a strong candidate for a male-determining factor in Anopheles stephensi
Source: eLife. 2016 Sep 20;5:e19281. doi: 10.7554/eLife.19281 (PMC5061544; doi:10.7554/eLife.19281)
Supplement: Figure 4—source data 1. — DOI: http://dx.doi.org/10.7554/eLife.19281.010 [file elife-19281-fig4-data1.docx]

**Figure 4-source data 1.** Assay for male reproductive competitiveness of *nGuy1-1* and *nGuy1-2* lines ^1^. This table contains the source data for Figure 4.

|  | nGuy1-1 Male No. (P) ^2, 3^ | nGuy1-1 female No. (P) ^2^ | non-transgenic male No. (P) ^2^ | non-transgenic  female No. (P) ^2^ | | Total of all genotypes | |
| --- | --- | --- | --- | --- | --- | --- | --- |
| Replicate 1 | 43 (24.57%) | 0 (0) | 62 (35.43%) | 70 (40%) | | 175 | |
| Replicate 2 | 38 (31.15%) | 0 (0) | 36 (29.51%) | 48 (39.34%) | 122 | |  |
| Replicate 3 | 47 (15.67%) | 0 (0) | 122 (40.67%) | 131 (43.67%) | | 300 | |

|  | nGuy1-2 Male No. (P) ^2, 3^ | nGuy1-2 female No. (P) ^2^ | non-transgenic male No. (P) ^2^ | non-transgenic  female No. (P) ^2^ | | Total of all genotypes | |
| --- | --- | --- | --- | --- | --- | --- | --- |
| Replicate 1 | 130 (30.63%) | 0 (0) | 153 (37.23%) | 128 (31.14%) | | 411 | |
| Replicate 2 | 33 (15.87%) | 0 (0) | 81 (38.94%) | 94 (45.19%) | 208 | |  |
| Replicate 3 | 75 (18.16%) | 0 (0) | 170 (41.16%) | 168 (40.68%) | | 413 | |

Notes:

1. Methods of the assay: Sibling cohorts of 20 transgenic and 20 non-transgenic males were mated with 10 wild type females. The resulting progeny were screened for the dsRED marked and sexed at L3 instar stage.
2. No. (P): Number (No.) and proportion or percentage (P) of individuals of a certain genotype. The percentage (P) is calculated by dividing the observed number of individuals of a genotype by the total of all genotypes during each biological replicates. For example, there are (43+62+70)=175 total progeny in replicate 1 for the *nGuy1-1* line. Therefore, the percentage of transgenic males (A^GUY1^aXY) is 43/175=24.57%.
3. The expected proportion or percentage of each genotype in the progeny can be calculated according to the following. Transgenic A^GUY1^aXY mate with aaXX will produce A^GUY1^aXY, A^GUY1^aXX, aaXY, and aaXX at a 1:1:1:1 ratio. Non-transgenic aaXY mate with aaXX will produce aaXY, aaXX, aaXY, and aaXX at a 1:1:1:1 ratio. Assuming that A^GUY1^aXY and aaXY males are reproductively equal, the ratio of progeny from females mated with equal number of A^GUY1^aXY and aaXY males will be A^GUY1^aXY:A^GUY1^aXX: aaXY:aaXX= 1:1 :(1+2): (1+2)=1:1:3:3. Given that A^GUY1^aXX dies prior to or soon after egg hatching, at L3 instar, A^GUY1^aXY will be expected to represent 1/(1+3+3) or 1/7 of the total progeny, while aaXY and aaXX will represent 3/7 each. The percentage of DsRed positive (or transgenic) male progeny is significantly higher than the expected value (1/7, or 14.29%). This is confirmed by one-sample proportion tests for both *nGuy1-1* and *nGuy1-2* lines (Z=5.0 and 8.1, respectively; p<0.001 in both cases). Percentage data shown in this column are used to graph Figure 4.
