# Supplementary material for: GUY1 confers complete female lethality and is a strong candidate for a male-determining factor in Anopheles stephensi
Source: eLife. 2016 Sep 20;5:e19281. doi: 10.7554/eLife.19281 (PMC5061544; doi:10.7554/eLife.19281)
Supplement: Supplementary file 1. — DOI: http://dx.doi.org/10.7554/eLife.19281.012 [file elife-19281-supp1.docx]

**Supplemental file 1.** Number of male and female transgenics (DsRed positive) in the *nGuy1* and *Guy1m* lines.

**Supplemental file 1a.** Number of male and female transgenics (DsRed positive) in the *nGuy1* lines.

| **nGuy1 Lines (before split)** | **DsRed male** | **DsRed female** |
| --- | --- | --- |
| G2 | 134 | 0 |
| G3 | 35 | 0 |
| **Split into 2, nGuy1-1** |  |  |
| G4 | 218 | 0 |
| G5 | 91 | 0 |
| G6 | 107 | 0 |
| G7 | 75 | 0 |
| G8 | 145 | 0 |
| G9-15 | 635 | 0 |
| **Split into 2, nGuy1-2** |  |  |
| G4 | 11 | 0 |
| G5 | 62 | 0 |
| G6 | 27 | 0 |
| G7 | 129 | 0 |
| G8 | 87 | 0 |
| G9-15 | 269 | 0 |
| Total of the two lines, G2-G15 | 2025 | 0 |
| *nGuy1-1* numbers from Table S3 (L4 only ) | 300 | 0 |
| *nGuy1-1* numbers from Table S4 | 128 | 0 |
| *nGuy1-2* numbers from Table S4 | 208 | 0 |
| **Grand total** | **2661** | **0** |

**Supplemental file 1b.** Number of male and female transgenics (DsRed positive) in the *Guy1m* line.

| **Guy1m line** | **DsRed male** | **DsRed female** |
| --- | --- | --- |
| G2 | 152 | 207 |
| G3 | 73 | 91 |
| G4 | 51 | 55 |
| G5 | 30 | 34 |
| G6 | 21 | 28 |
| G7 | 69 | 62 |
| G8 | 24 | 28 |
| **Total** | **420** | **505** |

Note: Numbers were not recorded for *Guy1m* after G8.
