# Supplementary material for: GUY1 confers complete female lethality and is a strong candidate for a male-determining factor in Anopheles stephensi
Source: eLife. 2016 Sep 20;5:e19281. doi: 10.7554/eLife.19281 (PMC5061544; doi:10.7554/eLife.19281)
Supplement: Supplementary file 2. — DOI: http://dx.doi.org/10.7554/eLife.19281.013 [file elife-19281-supp2.docx]

**Supplemental file 2.** Number of transgenic (DsRed positive) males in the *bGuy1C* and *bGuy1N* lines.

| Line | G2-RH* | | G3-RH* | G4-RH* | G4-RS** | G5-G12*** |
| --- | --- | --- | --- | --- | --- | --- |
| *bGuy1N-1* | 33 | | 36 | 38 | 77 | 546 |
| *bGuy1N-2* | 10 | | 2 | 17 | 78 | 488 |
| *bGuy1N-3* | 40 | | 43 | 51 | 56 |  |
| *bGuy1N-4* | 28 | | 34 | 40 | 34 |  |
| *bGuy1N-5* | 14 | | 32 | 41 | 40 |  |
| *bGuy1N-6* | 28 | | 31 | 34 | 38 | 382 |
| *bGuy1N-7* | 37 | | 31 | 35 | 30 |  |
| *bGuy1N-8* | 23 | | 36 | 30 | 38 |  |
| *bGuy1N-9* | 10 | | 29 | 31 | 82 |  |
|  |  | |  |  |  |  |
| **Grand total** |  | |  |  |  | **2703** |
|  |  | |  |  |  |  |
| *bGuy1C-1* | 33 | | 36 | 46 | 78 | 455 |
| *bGuy1C-2* | 8 | | 33 | 24 | 57 | 386 |
| **Grand total** | |  |  |  |  | **1156** |

* Number of DsRed positive larvae screened to keep the line going. **All DsRed positives are males.** Numbers are from Rob Harrel from the University of Maryland Insect Transgenic Facility.

** Number of DsRed positive larvae screened to keep the line going. **All DsRed positives are males.** Numbers are from Randy Saunders at Virginia Tech, who performed an independent screening.

*** Only three *bGuy1N* lines and two *bGuy1C* lines were kept beyond G5.
