# Supplementary material for: GUY1 confers complete female lethality and is a strong candidate for a male-determining factor in Anopheles stephensi
Source: eLife. 2016 Sep 20;5:e19281. doi: 10.7554/eLife.19281 (PMC5061544; doi:10.7554/eLife.19281)
Supplement: Supplementary file 3. — DOI: http://dx.doi.org/10.7554/eLife.19281.014 [file elife-19281-supp3.docx]

**Supplemental file 3.** Supplemental sequences.

**Inverse PCR**

>*nGuy1-1*, Insertion site matches scaffold_00109

ATTACTGAGATGTCCTAAATGCACAGCGACGGATTCGCGCTATTTAGAAAGA

GAGAGCAATATTTCAAGAATGCATGCGTCAATTTTACGCAGACTATCTTTCTAGGGTTAA

TGAATGCGTCCTTCGGCTTCGGAGAGGAACGACGTCGCTTAGGAACCGGGAAGCCCTCGC

CCTCGAAACCGCTGGGCGCGGTGGTCACGGTGAGCACGGGACGTGCGACGGCGTCGGCGG

GTGCGGATACGCGGGGCAGCGTCAGCGGGTTCTCGACGGTCACGGCGGGCATGTCGACAG

ATCTGACAATGTTCAGTGCAGAGACTCGGA

>*nGuy1-2*, Insertion site matches scaffold_00082

ACTGAGATGTCCTAAATGCACAGCGACGGATTCGCGCTATTTAGAAAGAGGTGCTAAGAACCACCAACAGT

GATCAGGGTTGGAAAGCAAATGCACGCATCGAGTTTTGAAACTCCTAAGAAAACATCGTA

AATCGGCTTTAGACCGAAAGCACTCGCCCGAACCTCCCCAAATCGTCCGTTCTTGTTAGG

GGGCCGTTTTTTTGTCGTTGTTTTGTTTCTGTTTACAACGAGTGCCCATTTTCACGCAAT

CGGTGCAAACGGCGCAAGCAACAAAACTGGTGGAAAATTATTTCGTCACCCGGGAAGCCC

TCGCCCTCGAAACCGCTGGGCGCGGTGGTCACGGTGAACACGGGACGTGCGACGGCGTCG

GCGGGTGCGGATACGCGGGGCAGCGTCAGCGGGTTCTCGACGGTCACGGCGGGCATGTCG

ACAGATCTGACAATGTTCAGTGCAGAGACTCGGA

>Guy1m, Flanking sequence is too short to conclusively locate the insertion site.

ATTCCGAGTCTCTGCACTGAACATTGTCAGATCTGTCGACATGCCCGCCGTGACCGTCGAGAACCCGCTGACGCTGCCCCGCGTATCCGCACCCGCCGACGCCGTCGCACATCCCGTGCTCACCGTGACCACCGCGCC

CAGCGGTTTCGAGGGCGAGGGCTTCCCGGATGTCGCGCACCGCGTTTTTAACCCTAGAAA

GATAGTCTGCGTAAAATTGACGCATGCATTCTTGAAATATTGCTCTCTCTTTCTAAATAG

CGCGAATCCGTCGCTGTGCATTTAGGACATCTCAGTA

**Synthesis of the bGuy1C and bGuy1N constructs**

>bGuy1C Guy1_CDS_CTag_EE1

AAAAGCCTAGGCAAATTATGAATTCACAAAGTAGGCGATACAAAAACATTGAATTAGTGA

ATAATCTGAAAGCTTATCTGACTTGGAATGATAAATCAAGTTTTCAGGTTAAACACTCTG

CTGTGACATTGGAAAAAAAGAAAAGTAAAACAAAAATATGTAACGTACTCTATGAAGCAA

TCACAGGTGGAGGTGGTGGAGGTGGTGGTTGGTCACACCCCCAATTTGAAAAATAATTTT

TCCTCAGCGCC

>bGuy1N Guy1_CDS_Ntag_EE1no

AAAAGCCTAGGATGTGGTCACACCCCCAATTTGAAAAAGGTGGAGGTGGTGGAGGTGGTG

GTATGAATTCACAAAGTAGGCGATACAAAAACATTGAATTAGTGAATAATCTGAAAGCTT

ATCTGACTTGGAATGATAAATCAAGTTTTCAGGTTAAACACTCTGCTGTGACATTGGAAA

AAAAGAAAAGTAAAACAAAAATATGTAACGTACTCTATGAAGCAATCACATAAATAGTTC

ACAACGAAAAAAAGGCACATTTAGCATATCTGAAAATAAAACGTGACAGACAATAGCATA

AGACCACTTTGTGTGTTAATATAGTAAAATAGAGGCTATGGTGTCAATTTTTAACGGATC

GACTTAGTTTTTTTTTTGTAAAATTTAGTTTCACATGTTGGAAAAACGTTTCTTAATTAA

GTCGA
